# Indigenous well-being in four countries: An application of the UNDP'S Human Development Index to Indigenous Peoples in Australia, Canada, New Zealand, and the United States

**DOI:** 10.1186/1472-698X-7-9

**Published:** 2007-12-20

**Authors:** Martin Cooke, Francis Mitrou, David Lawrence, Eric Guimond, Dan Beavon

**Affiliations:** 1 Department of Sociology and the Department of Health Studies and Gerontology, University of Waterloo, 200 University Drive W, Waterloo, Ontario, Canada; 2 Centre for Developmental Health, Curtin University of Technology, Telethon Institute for Child Health Research, PO Box 855, West Perth. WA. 6872. Australia; 3 Strategic Research and Analysis Directorate, Indian and Northern Affairs Canada, Terrasses de la Chaudière, 10 Wellington, North Tower, Gatineau, Quebec, Canada; 4 Department of Sociology, University of Western Ontario, London, Canada

## Abstract

**Background:**

Canada, the United States, Australia, and New Zealand consistently place near the top of the United Nations Development Programme's *Human Development Index (HDI) *rankings, yet all have minority Indigenous populations with much poorer health and social conditions than non-Indigenous peoples. It is unclear just how the socioeconomic and health status of Indigenous peoples in these countries has changed in recent decades, and it remains generally unknown whether the overall conditions of Indigenous peoples are improving and whether the gaps between Indigenous peoples and other citizens have indeed narrowed. There is unsettling evidence that they may not have. It was the purpose of this study to determine how these gaps have narrowed or widened during the decade 1990 to 2000.

**Methods:**

Census data and life expectancy estimates from government sources were used to adapt the Human Development Index (HDI) to examine how the broad social, economic, and health status of Indigenous populations in these countries have changed since 1990. Three indices – life expectancy, educational attainment, and income – were combined into a single HDI measure.

**Results:**

Between 1990 and 2000, the HDI scores of Indigenous peoples in North America and New Zealand improved at a faster rate than the general populations, closing the gap in human development. In Australia, the HDI scores of Indigenous peoples decreased while the general populations improved, widening the gap in human development. While these countries are considered to have high human development according to the UNDP, the Indigenous populations that reside within them have only medium levels of human development.

**Conclusion:**

The inconsistent progress in the health and well-being of Indigenous populations over time, and relative to non-Indigenous populations, points to the need for further efforts to improve the social, economic, and physical health of Indigenous peoples.

## Background

As of January, 2007, we were two years into the second United Nations International Decade of the World's Indigenous Peoples. This may be a surprise, as this type of pronouncement of the importance of the rights and equality of the world's Indigenous peoples tends to capture media attention for only a short time before fading from the headlines. The average conditions of Indigenous peoples are generally well below national levels and disparities between Indigenous and non-Indigenous populations in health, social, and economic outcomes exist worldwide, in rich and poor countries alike, despite widely differing geographic, historical, and cultural contexts [1,2]. Among highly-developed countries, Canada, the United States, Australia, and New Zealand are often seen as natural comparators in terms of Indigenous well-being. These countries consistently place near the top of the United Nations Development Programme's *Human Development Index (HDI) *rankings, yet all have minority Indigenous populations with much poorer health and social conditions than their non-Indigenous compatriots [3]. First Nations (Registered non-status Indians), Métis and Inuit in Canada, Australian Aboriginal and Torres Strait Islander peoples, New Zealand Māori, and American Indians and Alaska Natives in the U.S. have each been subjected to loss of culture, paternal protectionism, and occasional violence that have characterized Indigenous-settler state relations in these former British colonies [4,5].

In the past thirty years, Indigenous populations in these countries have recovered from the very high mortality rates seen over much of the 19^th ^century. Although there are differences in morbidity and the major causes of mortality in these populations, there are also considerable similarities. They have mainly passed through demographic and epidemiological transitions whereby infectious diseases, although still much more prevalent than in the mainstream societies, have declined as causes of death, and mortality is now increasingly related to lifestyle or man-made causes [6]. Rates of smoking are high, as are rates of alcoholism and substance abuse. Obesity and Type II diabetes are now major health problems in each of these populations, as are deaths due to suicide, accidents and violence [7-9]. Clearly, these poor health conditions are closely related to social and economic context. Indigenous peoples in each of these countries are more likely to be unemployed, to leave school early, and to live in poverty than are other citizens. This is particularly true for those who live in discrete Indigenous communities, but also the case for the growing proportion who live in other urban or rural areas [1].

In these countries, recent decades have seen changes in the relationships between Indigenous peoples and the state. Beginning in the 1960s, strong Indigenous rights movements were influential in bringing Indigenous issues to public attention and, although we cannot list all of them here, there have been important legislative changes and legal decisions affecting Indigenous rights. In Canada, Indigenous rights were included in the *Constitution Act *of 1982, and the Royal Commission on Aboriginal Peoples, which ran from 1991 to 1996, examined the social, economic, legal and health status of Indigenous peoples [10]. This prompted a statement of reconciliation from the Canadian government in which it acknowledged the role it played in the development and administration of Indian residential schools [11] and Canadian courts have recently approved payment and funding for programmes for former students and their families for healing, truth, reconciliation, and commemoration of the residential schools and the abuses suffered [12]. In addition, there have been in Canada important judicial decisions confirming Indigenous rights, such as the 1999 Marshall decision regarding fishing rights and the 2006 Gray decision regarding the right to harvest wood on Crown lands for domestic uses [13]. In the U.S., tribes were granted new taxation powers in the 1980s, allowing them to better fund their own social programs and, as in Canada, there has been devolution of control over health and social services to Indigenous communities since the mid-1980s [5].

In Australia, Indigenous peoples were granted rights to equal pay in 1965, and a 1967 referendum transferred some powers in respect to Indigenous peoples from the states to the federal government, leading to the establishment of an Indigenous representative body, the Aboriginal and Torres Strait Islander Commission (ATSIC). In 1992, the High Court decision in the Mabo case recognised the native title rights of Aboriginal Australians, and the 1993 *Native Title Act *established a legal basis for land claims. In New Zealand, the *Treaty of Waitangi Act *was amended in 1985 to strengthen the mandate of the Waitangi Claims Tribunal to hear claims of historical breaches of the treaty, and the 1993 *Māori Land Law Act *strengthened Māori land claims. New Zealand is the only one of the four countries in which there are a number of dedicated parliamentary seats for Indigenous people, and this number of Māori seats was increased in 1995 [4].

There have been some recent indications of a possible retrenchment of Indigenous rights. Australia has recently dissolved the ATSIC, and amalgamated government services to Indigenous communities into mainstream government agencies. The New Zealand parliament is currently considering a bill that would set September 1, 2008 as the cut-off date for the lodgement of historical treaty claims. However, in general, the recent changes in all of these countries can be seen as part of a slow and often contentious shift towards increasing the rights of Indigenous peoples and communities, including control over the provision of health and social services, although stopping well short of Indigenous self-determination [5,14].

Despite these changes, it remains generally unknown whether the overall conditions of Indigenous peoples are improving and whether the gaps between Indigenous peoples and other citizens have indeed narrowed in recent decades. There is unsettling evidence that they may not have. For example, advances in infant mortality among Indigenous people in Western Australia have not kept up with the non-Indigenous Australian population [15]. In Canada, there is evidence that the difference in average income between Registered Indians and other Canadians was rising in the late 1990s [16]. In this paper we examine how the broad social, economic, and health status of Indigenous populations in these countries have changed since the 1990s, using an adaptation of the United Nations' Human Development Index (HDI).

## Methods

### The Human Development Index

The United Nations Development Program (UNDP)'s HDI has been used since 1990 to compare countries in terms of "human development", defined as the enlargement of choices made possible by education and literacy, a decent material standard of living, and a long and healthy life. The HDI was created as an alternative to using GDP per capita to capture economic and social development. It was recognised that while GDP measures economic growth it does not adequately reflect the degree to which national product translates into the well-being of a population, and that other dimensions should be considered [17]. As the HDI was designed to measure all countries, and as many developing countries have limited national statistical collection capacity, it was necessary to balance the theoretical completeness of the index with practical issues of data availability. Therefore, the concept of human development was defined by the UNDP to include three broad and inter-related dimensions: an income sufficient to ensure a minimal material standard of living; knowledge, which is necessary for full participation in society; and health, which is a fundamental prerequisite to well-being. Health is measured using life expectancy, knowledge is measured via educational participation and adult literacy rates, and the material standard of living is captured by GDP per capita, reported in Purchasing Power Parity (PPP) dollars. These three indicators are combined, with equal weighting, to give an overall HDI score.

It should be noted that the HDI is one of a number of measures presented annually in the UNDP's *Human Development Report*. These include the Human Poverty Index, which is calculated separately for wealthy countries and for less developed ones, as well as indicators that take into account the gendered aspects of development [3]. There are also many other composite indicators of overall health and social development beyond the UNDP's measures, most requiring much better data than are available for the populations in the present study. However, the simple three-dimension HDI has become an important and widely-cited measure in the field of development studies, and has been credited with helping to widen the focus of development studies and raising the profile of important policy issues in a number of countries [14]. Although it captures only selected aspects of "well-being" or quality of life, the HDI has become part of the international development discourse.

In the case of Indigenous populations, previous application of a modified HDI to Canadian Registered Indians and Inuit found that there was far from consistent improvement in overall well-being between 1981 and 2001 [16,18]. Whereas it had been assumed that the well-being of Indigenous peoples was improving, relative to other Canadians, that research found disparities widened in some periods [16]. The purpose of this study is to compare Canada to other, similar countries in terms of the trends in well-being of their Indigenous populations, relative to other inhabitants. We focus on these four English-speaking countries because of their common history as British colonies, similarly high levels of overall human development, and similar overall systems of government and state provision. At the same time, there are some important differences among them in terms of Indigenous-state relationships [4]. Although the conditions of Indigenous populations in these countries are the result of various historic, economic, geographic, and political circumstances, this comparison allows us to judge whether the trends in Canada are unique, and give us some indication of whether the status of Indigenous peoples has generally improved, at least in these countries.

### Data sources

Following the methodology used to construct the modified HDI for Canadian Indigenous populations [16,18] we used (a) income and education data extracted from censuses and (b) life expectancy estimates derived from vital statistics to compare the well-being of Indigenous and non-Indigenous populations in Australia, Canada, New Zealand and the United States in the 1990s [19-32]. The populations included were Canadians who identified themselves as members of an Aboriginal group (North American Indian, Inuit, or Métis), those who reported having American Indian or Alaskan Native race in the 1990 and 2000 US censuses, and those identifying as being of Māori ethnicity in New Zealand, and being of Aboriginal or Torres Strait Islander origin in the Australian census. No institutional ethics approval was required for the use of these publicly-available aggregated data.

The detailed method of calculating a modified HDI to compare these populations is presented in Table 1. Median income in Purchasing Power Parity dollars [32], rather than GDP per capita, is used to measure the material standard of living. As a proxy for functional literacy, we used the proportion of the population aged 15 and older with grade nine or higher education in Canada or the US, with Year 9 or higher in Australia, with Sixth form or higher in New Zealand. Educational participation is captured by the proportion of the 18–24 year-old population who have completed secondary school, or some post-secondary, trades, or technical training.

**Table 1:** HDI index calculation

		*Measure*	*Min*	*Max*	*Index Formula*
*Education Index*	Adult Literacy (1/3)	Proportion 15 and older with grade 9 or higher education	0	1.0	ILiteracy=Xactual−Xmin⁡Xmax⁡−Xmin⁡ MathType@MTEF@5@5@+=feaagaart1ev2aaatCvAUfKttLearuWrP9MDH5MBPbIqV92AaeXatLxBI9gBaebbnrfifHhDYfgasaacPC6xNi=xH8viVGI8Gi=hEeeu0xXdbba9frFj0xb9qqpG0dXdb9aspeI8k8fiI+fsY=rqGqVepae9pg0db9vqaiVgFr0xfr=xfr=xc9adbaqaaeGacaGaaiaabeqaaeqabiWaaaGcbaGaemysaK0aaSbaaSqaaiabdYeamjabdMgaPjabdsha0jabdwgaLjabdkhaYjabdggaHjabdogaJjabdMha5bqabaGccqGH9aqpjuaGdaWcaaqaaiabdIfaynaaBaaabaGaemyyaeMaem4yamMaemiDaqNaemyDauNaemyyaeMaemiBaWgabeaacqGHsislcqWGybawdaWgaaqaaiGbc2gaTjabcMgaPjabc6gaUbqabaaabaGaemiwaG1aaSbaaeaacyGGTbqBcqGGHbqycqGG4baEaeqaaiabgkHiTiabdIfaynaaBaaabaGagiyBa0MaeiyAaKMaeiOBa4gabeaaaaaaaa@5560@
	Education (2/3)	Proportion 18–24 with High school or some post-secondary education	0	1.0	IEducation=Xactual−Xmin⁡Xmax⁡−Xmin⁡ MathType@MTEF@5@5@+=feaafiart1ev1aaatCvAUfKttLearuWrP9MDH5MBPbIqV92AaeXatLxBI9gBaebbnrfifHhDYfgasaacPC6xNi=xH8viVGI8Gi=hEeeu0xXdbba9frFj0xb9qqpG0dXdb9aspeI8k8fiI+fsY=rqGqVepae9pg0db9vqaiVgFr0xfr=xfr=xc9adbaqaaeGacaGaaiaabeqaaeqabiWaaaGcbaGaemysaK0aaSbaaSqaaiabdweafjabdsgaKjabdwha1jabdogaJjabdggaHjabdsha0jabdMgaPjabd+gaVjabd6gaUbqabaGccqGH9aqpjuaGdaWcaaqaaiabdIfaynaaBaaabaGaemyyaeMaem4yamMaemiDaqNaemyDauNaemyyaeMaemiBaWgabeaacqGHsislcqWGybawdaWgaaqaaiGbc2gaTjabcMgaPjabc6gaUbqabaaabaGaemiwaG1aaSbaaeaacyGGTbqBcqGGHbqycqGG4baEaeqaaiabgkHiTiabdIfaynaaBaaabaGagiyBa0MaeiyAaKMaeiOBa4gabeaaaaaaaa@56A7@
*Income Index*		Median total income for those 15 and older	PPP$100	PPP$40,000	IIncome=log(X)−log(Xmin)log(Xmax)−log(Xmin) MathType@MTEF@5@5@+=feaagaart1ev2aaatCvAUfKttLearuWrP9MDH5MBPbIqV92AaeXatLxBI9gBaebbnrfifHhDYfgasaacPC6xNi=xH8viVGI8Gi=hEeeu0xXdbba9frFj0xb9qqpG0dXdb9aspeI8k8fiI+fsY=rqGqVepae9pg0db9vqaiVgFr0xfr=xfr=xc9adbaqaaeGacaGaaiaabeqaaeqabiWaaaGcbaGaemysaK0aaSbaaSqaaiabdMeajjabd6gaUjabdogaJjabd+gaVjabd2gaTjabdwgaLbqabaGccqGH9aqpjuaGdaWcaaqaaiabbYgaSjabb+gaVjabbEgaNnaabmaabaGaemiwaGfacaGLOaGaayzkaaGaeyOeI0IaeeiBaWMaee4Ba8Maee4zaC2aaeWaaeaacqWGybawdaWgaaqaamXvP5wqSXMqHnxAJn0BKvguHDwzZbqegeKCPfgBaGabaiaa=1gacaWFPbGaa8NBaaqabaaacaGLOaGaayzkaaaabaGaeeiBaWMaee4Ba8Maee4zaC2aaeWaaeaacqWGybawdaWgaaqaaiaa=1gacaWFHbGaa8hEaaqabaaacaGLOaGaayzkaaGaeyOeI0IaeeiBaWMaee4Ba8Maee4zaC2aaeWaaeaacqWGybawdaWgaaqaaiaa=1gacaWFPbGaa8NBaaqabaaacaGLOaGaayzkaaaaaaaa@6667@
*Life Expectancy Index*		Life expectancy at birth	85 years	25 years	ILEB=Xactual−Xmin⁡Xmax⁡−Xmin⁡ MathType@MTEF@5@5@+=feaagaart1ev2aaatCvAUfKttLearuWrP9MDH5MBPbIqV92AaeXatLxBI9gBaebbnrfifHhDYfgasaacPC6xNi=xH8viVGI8Gi=hEeeu0xXdbba9frFj0xb9qqpG0dXdb9aspeI8k8fiI+fsY=rqGqVepae9pg0db9vqaiVgFr0xfr=xfr=xc9adbaqaaeGacaGaaiaabeqaaeqabiWaaaGcbaGaemysaK0aaSbaaSqaaiabdYeamjabdweafjabdkeacbqabaGccqGH9aqpjuaGdaWcaaqaaiabdIfaynaaBaaabaGaemyyaeMaem4yamMaemiDaqNaemyDauNaemyyaeMaemiBaWgabeaacqGHsislcqWGybawdaWgaaqaaiGbc2gaTjabcMgaPjabc6gaUbqabaaabaGaemiwaG1aaSbaaeaacyGGTbqBcqGGHbqycqGG4baEaeqaaiabgkHiTiabdIfaynaaBaaabaGagiyBa0MaeiyAaKMaeiOBa4gabeaaaaaaaa@4DDF@
*HDI*					IHDI=[ILEB+(13ILiteracy+23IEducation)+IIncome]3 MathType@MTEF@5@5@+=feaagaart1ev2aaatCvAUfKttLearuWrP9MDH5MBPbIqV92AaeXatLxBI9gBaebbnrfifHhDYfgasaacPC6xNi=xH8viVGI8Gi=hEeeu0xXdbba9frFj0xb9qqpG0dXdb9aspeI8k8fiI+fsY=rqGqVepae9pg0db9vqaiVgFr0xfr=xfr=xc9adbaqaaeGacaGaaiaabeqaaeqabiWaaaGcbaGaemysaK0aaSbaaSqaaiabdIeaijabdseaejabdMeajbqabaGccqGH9aqpjuaGdaWcaaqaamaadmaabaGaemysaK0aaSbaaeaacqWGmbatcqWGfbqrcqWGcbGqaeqaaiabgUcaRmaabmaabaWaaSaaaeaacqaIXaqmaeaacqaIZaWmaaGaemysaK0aaSbaaeaacqWGmbatcqWGPbqAcqWG0baDcqWGLbqzcqWGYbGCcqWGHbqycqWGJbWycqWG5bqEaeqaaiabgUcaRmaalaaabaGaeGOmaidabaGaeG4mamdaaiabdMeajnaaBaaabaGaemyrauKaemizaqMaemyDauNaem4yamMaemyyaeMaemiDaqNaemyAaKMaem4Ba8MaemOBa4gabeaaaiaawIcacaGLPaaacqGHRaWkcqWGjbqsdaWgaaqaaiabdMeajjabd6gaUjabdogaJjabd+gaVjabd2gaTjabdwgaLbqabaaacaGLBbGaayzxaaaabaGaeG4mamdaaaaa@63F4@

### Data quality

Although censuses are the most reliable source of time-series data on these Indigenous populations, there are some problems with the comparability of populations between countries and between years, restricting our examination to the 1990s. These countries also collect ethnicity data somewhat differently. In the Canadian and Australian censuses, the Indigenous population refers to people who self-identify as Aboriginal on the Census form, but in Canada this question has changed somewhat between census years [16]. For New Zealand, the Māori census population includes those who indicated "Māori", in response to the ethnicity question, and this has also changed slightly between 1991 and 1996 [33]. In the United States, the Indigenous population includes people who responded that their "race" was American Indian or Alaska Native. This race question also changed slightly between the 1990 and 2000 censuses, in order to allow multiple write-in responses [34].

Median annual income for those aged 15 and older with income was also taken from the census data. Whereas the Canadian and American census data reported point estimates of income, the Australian and New Zealand census data provided fourteen income categories, requiring the calculation of a median from grouped data. Fortunately, the categories were of relatively small width so we can have some confidence in these estimates of median income. Nonetheless, it is recognized that using income as a measure of wealth may not fully reflect the material well-being of Indigenous people or the differences between Indigenous and non-Indigenous populations. It also does not incorporate traditional economic activities, such as hunting or trapping, and does not consider the population with no money income.

Adjustments for national price and currency levels do not take into account higher prices in many remote Indigenous communities. As well, some income difference between Indigenous and non-Indigenous populations may be due to the younger age structure of Indigenous populations in these countries, and improvements over time may therefore partly reflect changes in age structure, rather than real improvement.

The education question on the Australian census was changed in 2001. Before 2001, the focus was on the age the respondent left school rather than on the year level completed. Therefore, the proportion of the population aged 15 and older who left school at age 15 or older was used for 1991 and 1996, and 2001 values were extrapolated, assuming the same improvement between 1991 and 1996 and between 1996 and 2001. We present both the extrapolated figure and the new 2001 figure where appropriate.

Estimating Indigenous life expectancy is difficult, and the accuracy of life tables can be influenced by the quality of recording of Indigenous status within death registers and the total population counts. Resulting numerator-denominator bias can impact on life expectancy estimates, and changes in bias over time can impact gaps over time [35,36]. Indigenous life tables calculated from vital statistics data and published by official sources have been used for all four countries, and where the estimate years did not correspond with the census years, they were linearly interpolated. Life expectancy estimates used for the Canadian Indigenous population were for Registered Indians, the only population for which national estimates are available, and which represent about 57% of the Canadian Aboriginal population in the 2001 Census. For New Zealand, a change in the census ethnicity question affected the comparability of 1991 and later life tables [33]. For this reason, we have not used the 1991 Indigenous life tables for New Zealand, but have backcast the 1996 and 2001 data using linear extrapolation. The resulting 1991 estimates are similar to those published by Blakely *et al*., who identify some overestimation of Māori life expectancy within these tables. They report that although Māori life expectancy increased over the 1980s and 1990s, the gap with non-Māori, non-Pacific Islanders in New Zealand widened over the period, to nearly 10 years [36,37]. As well, Hill *et al*. suggest the gap in life expectancy is around 13 years for Aboriginal Australians, compared with the gap of over 20 years estimated using official life tables [38]. Note that using these revised estimates would not change the ranking of the countries presented below, nor seriously change the overall picture of changes in Indigenous well-being in these countries. We therefore chose to use the original New Zealand life tables, which are centred on the census years and show a slightly narrowing life expectancy gap, and the original Australian figures, which provide a series of estimates over the period in which we are interested. These results are presented below.

## Results and Discussion

### Population size

The United States has the largest Indigenous population, estimated at 4,119,300 in the 2000 Census. However, American Indians and Alaska Natives comprise only 1.5 percent of the total American population. In relative terms, the Māori population is the largest, accounting for 14 percent of the total New Zealand population (at 526,200 people in the 2001 census). Just over 2 percent of Australians (or 410,000 people) identified as being Aboriginal or Torres Strait Islander people in the 2001 census. In Canada, 976,301 people or 3.3 percent of residents self-identified as Aboriginal in the 2001 census.

### Life expectancy

**Figure 1:**
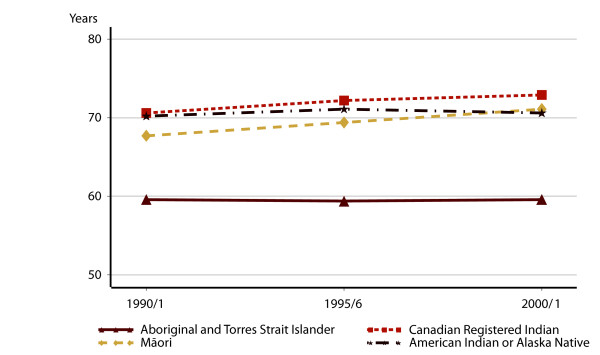
Life Expectance at birth, 1990/1–2000/1.

Improvements in Indigenous life expectancy and in closing the gap between Indigenous and non-Indigenous peoples varied among these four countries (Table 2). The national life expectancy estimates for the total population of each of the four countries in question was over 75 years throughout the 1990/1–2000/1 decade. Figure 1 shows life expectancy at birth from official estimates, for the four Indigenous populations of these countries over the same 10-year period. Canadian Registered Indians had the highest life expectancy of all of these populations, rising from 70.6 years to 72.9 years. Māori life expectancy also improved, from 69.4 years in 1996 to 71.1 years in 2001.

**Table 2:** Educational attainment proxy measures

Country	Adult Literacy Proxy	Gross enrolment proxy
Australia	1991, 1996: Proportion 15 and older that left school aged 15 years or older. 2001: Proportion 15 or older with highest educational qualification year 9 or higher.	1991, 1996: Proportion of those 18–24 still in school or left school aged 18 or older. 2001: Proportion of those 18–24 still in school, or with highest educational qualification Year 12 or equivalent.
Canada	Proportion 15 and older with grade nine or higher educational attainment.	Proportion of those 18–24 with secondary school certificate, some college, trades or technical, or university.
New Zealand	Proportion aged 15 and older with no school qualification.	Proportion aged 18–24 with sixth form or higher qualification.
United States	Proportion aged 15 and older with 9^th ^grade or higher educational attainment.	Proportion aged 18–24 with High school graduation, GED or higher educational attainment.

In Canada, the life expectancy gap reduced from 7.3 to 5.8 years over the decade. At the end of the period, the gap in life expectancy between Māori and non-Māori New Zealanders had declined only slightly, if at all, to 8.5 years in 2001. Australian Aboriginal people and Torres Strait Islanders stand out for having the lowest life expectancy, at 59.6 years in both 1991 and 2001. As life expectancy for non-Aboriginal Australians rose, the gap to Aboriginal people increased from 20.6 years to 23.2 years. American Indians and Alaska Natives began the period with a life expectancy of 70.2 years. This rose to 71.1 years in 1995/6, but fell again to 70.6 years in 2001, increasing the gap between Indigenous and non-Indigenous Americans from 5.2 years to 6.0 years (Table 3).

**Table 3:** Life expectancy at birth, years (Life expectancy index score)

	Australia Non-Aboriginal	Aboriginal and Torres Strait Islander	Aboriginal-Non-Aboriginal Gap
	
1990/1	80.2 (.920)	59.6 (.577)	20.6 (.343)
1995/6	81.4 (.939)	59.4 (.573)	22.0 (.366)
2000/1	82.8 (.964)	59.6 (.576)	23.2 (.388)
			
	Canada Non-Aboriginal	Canadian Aboriginal (Registered Indian)	Gap
	
1990/1	77.9 (.882)	70.6 (.760)	7.3 (.122)
1995/6	78.5 (.892)	72.2 (.787)	6.3 (.105)
2000/1	78.7 (.895)	72.9 (.798)	5.8 (.097)
			
	New Zealand Non-Aboriginal	Māori	Gap
	
1990/1^2^	76.4 (.856)	67.7 (.712)	8.7 (.144)
1995/6	78.0 (.883)	69.4 (.741)	8.6 (.142)
2000/1	79.6 (.910)	71.1 (.769)	8.5 (.141)
			
	United States Non-Aboriginal	American Indian and Alaska Native	Gap
	
1990/1	75.4 (.841)	70.2 (.753)	5.2 (0.88)
1995/6	76.2 (.854)	71.1 (.768)	5.1 (.086)
2000/1	76.6 (.859)	70.6 (.760)	6.0 (.099)

### Educational attainment

**Figure 2:**
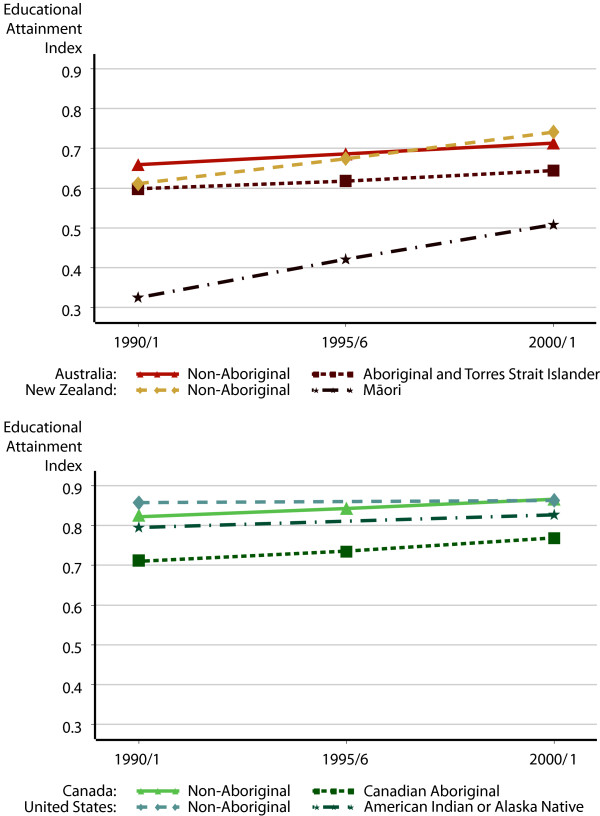
Education attainment index scores, 1990/1–2000/1.

Progress in educational attainment was slightly more evident. Figure 2 presents the Educational Attainment Index for the Indigenous and non-Indigenous populations in these countries. In the first panel, the consistent improvement in educational attainment for Māori is most striking, narrowing the gap with non-Māori. Although there was a smaller difference in educational attainment between Indigenous and non-Indigenous Australians, there is no evidence of this gap closing. Although the educational attainment of Aboriginal and Torres Strait Islanders increased, it did not keep pace with the improvements in the non-Indigenous population. The result was a slight widening of the gap in educational attainment.

Educational attainment scores were generally higher among North American populations. In the United States, the gap in the Educational Attainment Index was the smallest, and fell from 1990 to 2000. In Canada, the gap also narrowed between 1991 and 2001.

Table 4 shows how the educational attainment index is derived from the two component measures. In general, all four countries had high values on the adult literacy proxy measures, and the gaps between Indigenous and non-Indigenous populations improved between 1991 and 2001. The Māori population had the lowest proportion aged 15 and over with some basic school qualification, at about 57 percent in 2001, and the largest gaps between Indigenous and non-Indigenous people. However, these gaps declined considerably between 1991 and 2001, from 29 to 20 percentage points. In 2001, 83% of the Australian Aboriginal population 15 and older population had attained primary school or higher. The Canadian Indigenous population scored somewhat higher, and the American Indian and Alaska Native population had the highest adult literacy proxy scores, at .91 in 2001.

**Table 4:** Educational attainment measures, 1990/1 – 2000/1

	*Adult Literacy Proxy (2/3 weight)*			*Gross Enrolment Proxy (1/3 weight)*			*Educational Attainment Index*		
			
	Non-Aboriginal	Aboriginal	Gap	Non-Aboriginal	Aboriginal	Gap	Non-Aboriginal	Aboriginal	Gap
*Australia Aboriginal And Torres Strait Islander*									
1991	0.85	0.84	0.02	0.28	0.13	0.15	.659	.598	.061
1996	0.86	0.84	0.02	0.33	0.17	0.16	.686	.618	.068
2001	0.88	0.86	0.02	0.38	0.22	0.16	.713	.644	.069
2001*	0.91	0.83	0.07	0.69	0.31	0.38	.832	.659	.176
*Canada Aboriginal*									
1991	0.86	0.82	0.05	0.76	0.51	0.25	.826	.713	.113
1996	0.88	0.85	0.03	0.77	0.53	0.24	.843	.738	.105
2001	0.90	0.88	0.02	0.79	0.56	0.23	.866	.773	.093
*New Zealand Māori*									
1991	0.65	0.35	0.29	0.54	0.27	0.28	.611	.325	.286
1996	0.70	0.45	0.25	0.63	0.37	0.27	.674	.421	.253
2001	0.78	0.57	0.20	0.67	0.38	0.29	.741	.508	.233
*American Indian and Alaska Native*									
1990	0.90	0.88	0.03	0.77	0.63	0.13	.857	.795	.062
2000	0.92	0.91	0.02	0.75	0.67	0.08	.863	.827	.036

*Note*: Australian 1991–2001 figures are calculated using age at school-leaving. 2001* figures calculated using educational attainment.

Table 4 also presents the proportion of the population aged 18–24 with high school or higher education, our measure of educational participation. The attainment of all of the Indigenous populations improved considerably over the decade. However, in Australia and New Zealand this improvement did not keep pace with the increasing educational attainment among the non-Indigenous populations, and these countries saw the gaps widen between Indigenous and non-Indigenous populations.

### Median income

Although the educational attainment of Indigenous people increased over the decade, real incomes tended to fall over the 1990–2000 period. Median annual incomes from all sources for those aged 15 and over with income are presented in year 2000 Purchasing Power Parity Dollars in Table 5. In Australia, Canada, and New Zealand, real median incomes fell for the Indigenous and non-Indigenous populations between 1990 and 2000. In Canada and New Zealand, incomes fell between 1990 and 1995, rising somewhat thereafter, whereas Australian median incomes declined even more steeply between 1995 and 2001.

**Table 5:** Median annual income, 2000 PPP$ (Income index score)

	Australia Non-Aboriginal	Aboriginal and Torres Strait Islander	Gap
	
1990/1	25,795 (.927)	16,283 (.850)	9,512 (.077)
1995/6	25,579 (.925)	15,337 (.840)	10,242 (.085)
2000/1	21,767 (.898)	12,268 (.803)	9,499 (.095)
			
	Canada Non-Aboriginal	Canadian Total Aboriginal	Gap
	
1990/1	31,084 (.958)	19,970 (.884)	11,114 (.074)
1995/6	26,441 (.931)	16,931 (.857)	9,410 (.074)
2000/1	27,617 (.938)	18,713 (.873)	8,904 (.065)
			
	New Zealand Non-Aboriginal	Māori	Gap
	
1990/1	30,973 (.957)	23,936 (.914)	7,037 (.043)
1995/6	29,020 (.946)	22,838 (.906)	6,182 (.040)
2000/1	29,756 (.951)	23,024 (.908)	6,732 (.043)
			
	United States Non-Aboriginal	American Indian and Alaska Native	Gap
	
1990/1	19,372 (.879)	12,648 (.808)	6,724 (.071)
2000/1	21,050 (.893)	16,000 (.847)	5,050 (.046)

**Figure 3:**
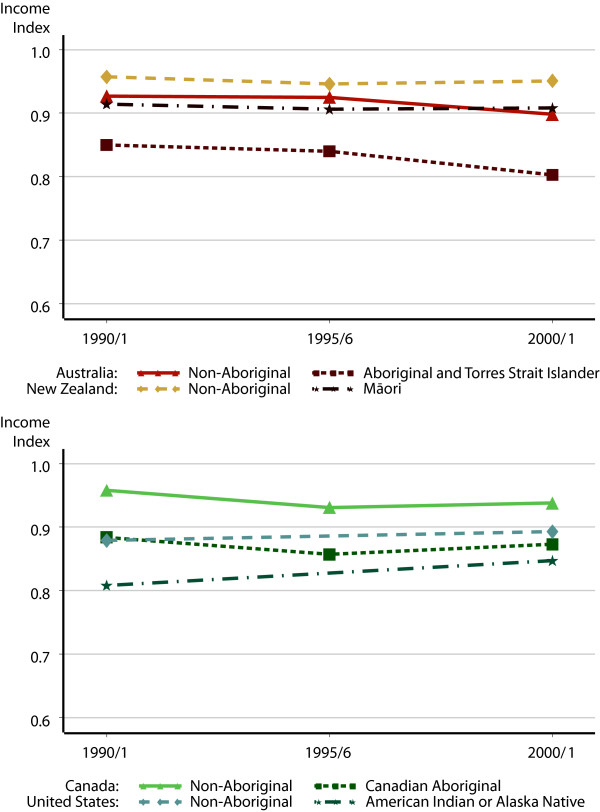
Income index scores, 1990/1–2000/1.

Figure 3 presents the Income index, calculated using median individual income. In all of these countries, the gap in income index scores between Indigenous and non-Indigenous citizens fell. In Canada, the effect of an economic recession in the early 1990s seems to have been greater for non-Indigenous than Indigenous Canadians, probably because of greater attachment to the labour force. The gap in income fell from PPP$11,114 to PPP$8,904 between 1990 and 2000, and the gap in income index scores fell from 0.074 to 0.065. In the United States, the gap also fell, from PPP$6,724 to PPP$5,050 in median income, and from 0.071 to 0.046 in terms of the Income Index.

Progress was again uneven in Australia. Over the 1990 to 2000 period, median incomes for both Indigenous and non-Indigenous Australians and New Zealanders declined. The difference between non-Aboriginal Australians and Aboriginal and Torres Strait Islanders was PPP$9,512 in 1990, rising to PPP$10,242 in 1995 and decreasing again to PPP$9,499 by 2000. Despite the fact that the gap in median income was the same at the beginning and end of the decade, the lower median income for both populations resulted in an increasing gap when calculated according the discounted income index formula. In New Zealand, where the gap between Māori and non-Māori incomes is smaller, the income index gap was the same at the beginning and end of the period.

### Indigenous Human Development Index scores

Following the methodology described earlier, these three indices – life expectancy, educational attainment, and income – are combined into a single HDI measure. Examining the trends in HDI scores, we see that the trends are somewhat different in these countries (Table 6). The HDI scores of American and Canadian Indigenous populations increased over the 1990 to 2000 decade at a faster rate than the non-Indigenous populations, closing the gap in human development. The difference between non-Indigenous and Indigenous Canadians fell from 0.103 in 1991 to 0.085 in 2001. In the United States, the gap decline was sharper, from 0.074 to 0.061 by 2000.

**Table 6:** Aboriginal Human Development Index Scores 1991 – 2001

	Australia Non-Aboriginal	Aboriginal and Torres Strait Islander	Aboriginal-Non-Aboriginal Gap
	
1990/1	.835	.675	.160
1995/6	.850	.677	.173
2000/1	.858	.674	.184
			
	Canada Non-Aboriginal	Canadian Aboriginal	Gap
	
1990/1	.889	.786	.103
1995/6	.889	.794	.095
2000/1	.900	.815	.085
			
	New Zealand Non-Aboriginal	Māori	Gap
	
1990/1^2^	.808	.650	.158
1995/6	.835	.689	.146
2000/1	.867	.728	.139
			
	United States Non-Aboriginal	American Indian and Alaska Native	Gap
	
1990/1	.859	.785	.074
2000/1	.872	.811	.061

*Note*: Australian 2001 figures calculated using extrapolated educational attainment (see text).

Māori HDI scores also improved at a faster rate than non-Māori New Zealanders'. The improvement in Māori HDI scores from 0.650 in 1991 to 0.728 in 2001 means a decrease in the HDI gap.

**Figure 4:**
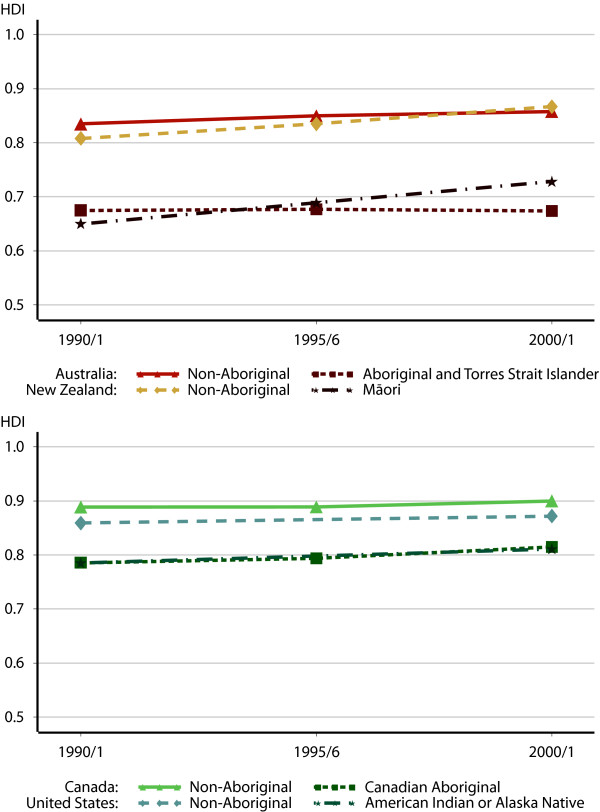
Human Development Index scores, 1990/1–2000/1.

While the gaps between Indigenous and non-Indigenous populations in these three countries closed over the decade, Australia stands out for a relative lack of progress on these indicators (Figure 4). The HDI score for Aboriginal and Torres Strait Islanders decreased slightly over the period, while the scores for non-Aboriginal Australians improved. The result is a widening gap in HDI scores, from 0.160 in 1990 to 0.184 by 2001.

### Indigenous populations – in an international perspective

These Indigenous HDI scores can be used to calculate positions for the four Indigenous populations in question among the countries ranked in the UNDP's 2003 *Human Development Report *(which also uses 2001 data) [3]. As described above, the measures used by the UNDP are not available for these populations. In order to make our figures closely comparable to the UNDP's measure, we adjusted each of our measures by the ratio of the UNDP's measure for the national population to our measure for the national population. Under the assumption that the ratio between, for example, the UNDP's adult literacy rate and our own census measure holds for both the Indigenous and non-Indigenous populations, we can use that ratio to estimate an HDI score for these Indigenous populations that is comparable to the countries in the *Human Development Index*.

As Table 7 shows, each of these four countries was well within the group of nations that the UNDP identifies as having "high human development". By 2001, the American Indian and Alaska Native population and the Canadian Indigenous population had joined these countries, with HDI scores comparable to South Korea or the Czech Republic and Belarus or Trinidad and Tobago, respectively. By 2001, Māori would rank around 73^rd ^among nations in the HDI league tables, among those countries with "medium" levels of human development. Australian Aboriginal and Torres Strait Islander peoples would rank approximately 104^th^, comparable to China and Cape Verde in terms of HDI score. This would also be classified among countries having "medium" levels of human development.

**Table 7:** Selected international and Aboriginal HDI scores, 2001

HDI Rank	Country	HDI Score
***Selected Countries with High Human Development (0.800–1)***		
1	Norway	.944
2	Iceland	.942
3	Sweden	.941
4	*Australia*	*.939*
5	Netherlands	.938
6	Belgium	.937
7	*United States*	*.937*
8	*Canada*	*.937*
9	Japan	.932
13	United Kingdom	.930
16	Austria	.929
17	France	.925
19	Spain	.925
20	*New Zealand*	*.917*
23	Portugal	.896
30	Republic of Korea	.879
	*U.S. American Indian and Alaska Native*	*.877*
32	Czech Republic	.861
	*Canadian Aboriginal Population*	*.851*
34	Argentina	.849
42	Costa Rica	.831
43	Chile	.831
52	Cuba	.806
53	Belarus	.804
54	Trinidad and Tobago	.802
55	Mexico	.800
***Selected Countries with Medium Human Development (0.500 – 0.799)***		
73	Saudi Arabia	.769
	*New Zealand Māori*	*.767*
75	Ukraine	.766
85	Philippines	.751
94	Dominican Republic	.737
103	Cape Verde	.727
	*Australian Aboriginal and Torres Strait Islanders*	*.724*
104	China	.721
105	El Salvador	.719
120	Egypt	.648
***Selected Countries with Low Human Development (0. – 0.499)***		
142	Cameroon	0.499
150	Haiti	0.467
161	Côte d'Ivoire	0.396

Source: UNDP (2003); Authors' Calculations

## Conclusion

It is acknowledged that these measures give only a very rough assessment of the degree to which the well-being of Indigenous peoples in these countries has improved. They necessarily hide a great deal of heterogeneity among these populations, and omit many other aspects of well-being or "human development". However, these simple indicators of life expectancy, educational attainment, and median income do give us a picture of the overall health and socioeconomic status of these populations, and how they have changed over the 1990s.

The resulting picture is best described as one of inconsistent progress. The improvement in overall HDI scores for Indigenous peoples in Canada, New Zealand, and the U.S. is good news, but the lack of progress in Australia is worrying. Furthermore, even in those countries in which the relative well-being of Indigenous populations did improve, as judged by their HDI scores the gaps on some indicators widened in some years. This suggests that further improvements in the social, economic, and physical health of Indigenous peoples cannot be taken for granted, and that further efforts must be made if we are to see these gaps close further by the end of this decade.

## Competing interests

The author(s) declare that they have no competing interests.

## Authors' contributions

MC and DB conceived of the original idea for the study. MC carried out the initial analysis, and drafted the manuscript. DB and EG assisted with the acquisition and analysis of the Canadian and US data used in the analysis, provided advice on methodology, and helped draft the manuscript. FM and DL assisted with the acquisition and analysis of the Australian and New Zealand data used in the analysis, assisted with the design of the methodology, and assisted with the drafting of the manuscript. All authors read and approved the final manuscript.

## Pre-publication history

The pre-publication history for this paper can be accessed here:



## References

[B1] Eversole R, Eversole R, McNeish JA, Cimadamore AD (2005). Overview: Patterns of Indigenous disadvantage worldwide. Indigenous peoples and poverty: an international perspective.

[B2] Stephens C, Porter J, Nettleton C, Willis R (2006). Disappearing, displaced, and undervalued: a call to action for Indigenous health worldwide. Lancet.

[B3] United Nations Development Program (2003). Human development report 2003.

[B4] Armitage A (1995). Comparing the policy of Aboriginal assimilation: Australia, Canada, and New Zealand.

[B5] Cornell S (2004). Indigenous jurisdiction and daily life: Evidence from North America. Paper presented at the National Forum on Indigenous health and the treaty debate.

[B6] Olshansky SJ, Ault B (1986). The fourth stage of the epidemiologic transition: The age of delayed degenerative diseases. The Milbank Quarterly.

[B7] Trovato F (2001). Aboriginal mortality in Canada, the United States and New Zealand. J Biosoc Sci.

[B8] Bramley D, Hebert P, Jackson R, Chassin M (2004). Indigenous disparities in disease-specific mortality, a cross-country comparison: New Zealand, Australia, Canada, and the United States. N Z Med J.

[B9] Hunter E, Harvey D (2002). Indigenous suicide in Australia, New Zealand, Canada, and the United States. Emerg Med (Fremantle).

[B10] Canada (1996). Report of the Royal Commission on Aboriginal Peoples. Ottawa.

[B11] Canada (1997). Gathering Strength: Canada's Aboriginal Action Plan. Ottawa.

[B12] Canada (2007). Residential Schools Settlement: Official Court Notice. Ottawa.

[B13] Supreme Court of Canada (2006). R. v. Sappier; R. v. Gray. Ottawa.

[B14] Lavoie J (2004). Governed by contracts: The development on Indigenous primary health services in Canada, Australia and New Zealand. J Abor Health.

[B15] Freemantle CJ, Read AW, de Klerk NH, McAullay D, Anderson IP, Stanley FJ (2006). Patterns, trends, and increasing disparities in mortality for Aboriginal and non-Aboriginal infants born in Western Australia, 1980–2001: population database study. Lancet.

[B16] Cooke M, Beavon D, McHardy M, White JP, Maxim P, Beavon D (2004). Measuring the well-being of Aboriginal People: An application of the United Nations Human Development Index to Registered Indians in Canada, 1981–2001. Aboriginal policy research: setting the agenda for change.

[B17] ul Haq M, Fukuda-Parr S, Shiva Kumar AK (2005). The human development paradigm. Readings in human development: Concepts, measures and policies for a developmental paradigm.

[B18] Senécal S, O'Sullivan E (2006). The Well-Being of Inuit Communities in Canada.

[B19] Australian Bureau of Statistics (1997). Occasional paper: Mortality of Aboriginal and Torres Strait Islander Australians. Cat no 33150 Canberra.

[B20] Australian Bureau of Statistics (1998). 1996 census of population and housing: Aboriginal and Torres Strait Islander people. Cat no 20340 Canberra.

[B21] Australian Bureau of Statistics (2002). Deaths Australia Cat no 33020 Canberra.

[B22] Statistics New Zealand (1999). New Zealand Life Tables (1995–1997).

[B23] Statistics New Zealand (2004). New Zealand life tables (2000–2002).

[B24] U.S. Bureau of the Census (1994). 1990 Census of Population-Subject Reports: Education in the US.

[B25] U.S. Bureau of the Census (2000). Census 2000 summary file 4.

[B26] Statistics Canada (1995). Life Tables, Canada and Provinces, 1990–92. Cat no 84-537 Ottawa.

[B27] Statistics Canada (1998). Life Expectancy: Abridged Life Tables, at Birth and Age 65, by Sex, for Canada, Provinces, Territories, and Health Regions. CANSIM table 102-0016 Ottawa.

[B28] Verma R, Michalowski M, Gauvin RP Abridged Life Tables for Registered Indians in Canada, 1976–80 to 1996–2000. Paper presented at the annual meeting of the Population Association of America, May 1–3 2003, Minneapolis.

[B29] Norris MJ, Kerr D, Nault F (1995). Projections of the Population with Aboriginal Identity in Canada, 1991–2016.

[B30] Department of Indian Affairs and Northern Development (1998). Population Projections of Registered Indians, 1996–2021.

[B31] Department of Indian Affairs and Northern Development (2003). Basic Departmental Data 2002.

[B32] Organization for Economic Co-Operation and Development Purchasing Power Parities Data. http://www.oecd.org/document/47/0,2340,en_2649_34357_36202863_1_1_1_1,00.html.

[B33] Statistics New Zealand Change in Ethnicity Question – 2001 Census of Populations and Dwellings. http://www2.stats.govt.nz/domino/external/web/prod_serv.nsf/htmldocs/Change+in+ethnicity+question+-+2001+Census+of+Population+and+Dwellings.

[B34] U.S. Bureau of the Census Racial and ethnic classifications used in census 2000 and beyond. http://www.census.gov/population/www/socdemo/race/racefactcb.html.

[B35] Alwaji S, Blakely T, Robson B, Atkinson J, Kiro C (2003). Unlocking the numerator-denominator bias III: adjustment ratios by ethnicity for 1981-1999 mortality data. The New Zealand Census-Mortality Study. N Z Med J.

[B36] Blakely T, Tobias M, Robson B, Ajwani S, Bonne M, Woodward A (2005). Widening ethnic mortality disparities in New Zealand 1981–99. Soc Sci Med.

[B37] Ajwani S, Blakely T, Robson B, Bonne M, Tobias M (2003). Decades of Disparity: Ethnic mortality trends in New Zealand 1980–1999.

[B38] Hill K, Barker B, Vos T (2007). Excess Indigenous mortality: Are Indigenous Australians more severely disadvantaged than other Indigenous populations?. Int J Epidemiol.

